# Preconditioning offers cardioprotection in hyperlipidemic rat hearts: possible role of Dopamine (D2) signaling

**DOI:** 10.1186/s12872-015-0071-8

**Published:** 2015-07-28

**Authors:** Varun Gupta, Rohit Goyal, Pyare Lal Sharma

**Affiliations:** Department of Pharmacology, School of Pharmaceutical Sciences, Shoolini University, Solan, HP 173212 India; Department of Pharmacology, Post Graduate Institute of Medical Education and Research (PGIMER), Chandigarh, India

**Keywords:** Dopamine, Preconditioning, Hyperlipidemia, Infarct

## Abstract

**Background:**

Ischemic preconditioning (IPC) induced cardioprotection has been reported to be blunted in hyperlipidemic subjects. Dopamine, via its D2 receptor signaling, appears to mimic the signaling cascade involved in myocardial preconditioning and is also involved in the inhibition of hyperlipidemia induced mediators. The present study was designed to investigate the possible involvement of D2 receptors in IPC and to see whether dopamine preconditioning can offer cardioprotection in hyperlipidemic rat hearts.

**Methods:**

Wistar albino rats were divided into 8 groups and fed on normal or high fat diet for 4 weeks. Hyperlipidemia was confirmed after 4 weeks by serum lipid estimations. Isolated perfused hearts were subjected to ischemic preconditioning or dopamine induced pharmacological preconditioning followed by 30-min ischemic insult and 60-min reperfusion. Clozapine was administered as D2 antagonist. Coronary perfusate (basal and post-ischemic) was collected for the estimations of LDH (Lactate dehydrogenase) and CKMB (Creatine kinase MB). Hearts were then removed and frozen for infarct size measurement.

**Results:**

A significant increase body weight, serum lipids except HDL was noted in high fat diet fed rats, as compared to normal rats. The level of LDH, CKMB in coronary effluent and infarct size were found to be decreased in preconditioned normal hearts, as compared to hearts treated with ischemia reperfusion. This effect was found to be blunted in hyperlipidemic animals. Dopamine (10 μM) alone and in combination with ischemic preconditioning significantly reduced the levels of LDH, CKMB and infarct size in hyperlipidemic hearts, as compared to preconditioned and non-preconditioned hyperlipidemic hearts. This effect was abolished significantly by Clozapine (D2 antagonist).

**Conclusion:**

The present study reveals possible involvement of D2 receptors in ischemic preconditioning and suggests that dopamine preconditioning may offer significant cardioprotection in hyperlipidemic rat hearts.

## Background

Ischemic Preconditioning (IPC) is a protective phenomenon which provides resistance to myocardium against ischemic insult after brief episodes of sub-lethal ischemia and reperfusion [1]. IPC requires intervention in coronary blood supply and is not profoundly acceptable in most clinical cases. The endogenous triggers to IPC are adenosine, bradykinin, angiotensin, prostaglandin and endothelin [2–4] which activate families of intracellular kinases: protein kinase C, protein kinase B, tyrosine kinase, MAPK and GSK-3β [5, 6]. The end effector stages include activation of sarcolemmal & mitochondrial K_ATP_ channels and mitochondrial permeability transition pore [7–9] thereby providing myocardial tolerance to ischemia, and cardioprotection. Pharmacological preconditioning is an alternative to IPC which mimics the cardioprotective effect of IPC using certain drugs [10]. However, the clinical application of preconditioning is limited because of the blunted cardioprotective response of preconditioning in hyperlipidemia [11].

Hyperlipidemia is a pathological state which is characterized by elevated levels of serum lipids and TGs, subsequently resulting atherosclerosis, coronary heart disease, hypertension, myocardial infarction and stroke. The clogged arteries (due to the deposition of lipids) and released endogenous cell mediators in hyperlipidemic state exaggerate the myocardial ischemic injury [12]. The release of mediators such as eNOS, nucleotidase, peroxynitrite, tetrahydrobiopterin, superoxide anion etc. is considered to be the cause of blunted cardioprotective response of preconditioning in hyperlipidemic subjects [13]. Therefore, exploration of a pharmacological cascade which mimics IPC and its effect in hyperlipidemic state is of prime importance to render preconditioning clinically applicable.

**Fig. 1:**
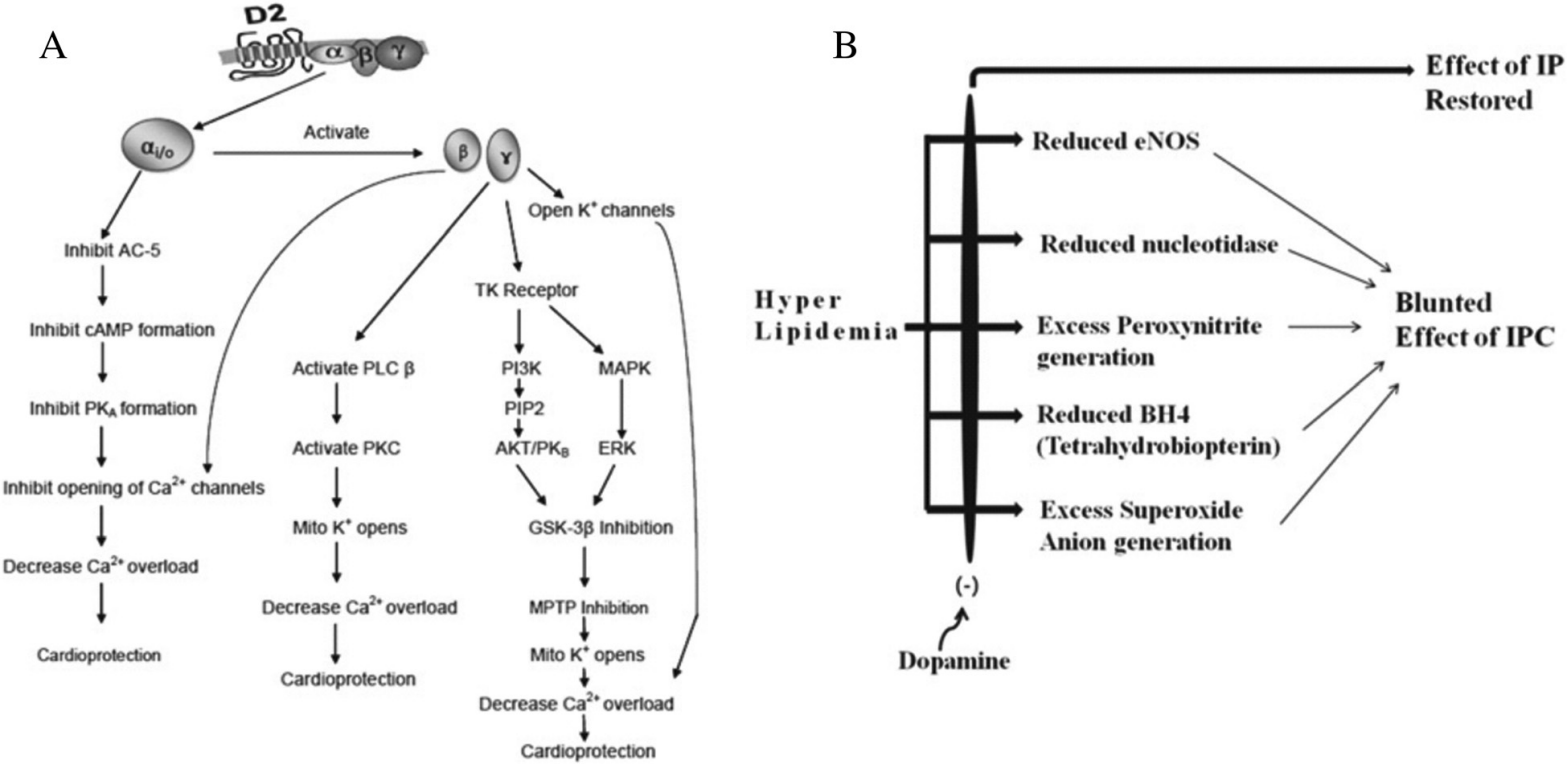
**(a)** Possible mechanism of Dopamine D2 receptor signaling in Ischemic Preconditioning; **(b)** Possible factors responsible for blunted effect of IPC in hyperlipidemia and reversible effect of Dopamine

Dopamine is a monoamine neurotransmitter having diverse effects on human physiology. It is used intravenously to treat congestive heart failure. The dopamine receptors are of 5 subtypes: D1, D2, D3, D4 and D5 which are metabotropic G protein-coupled receptors (GPCRs). All the 5 subtypes are reported to be present in human heart, except D3 [14]. The involvement of protein kinases, tyrosine kinase, GSK-3β, MPTP & MAPK has been observed in D2 receptor signaling [15, 16], and hence IPC mimicking effect of D2 signaling may be hypothesized (Fig. 1a). An *in-vitro* report revealed the involvement of D2 receptors in protection of cardiomyocytes against ischemia reperfusion induced apoptotic damage [17]. A report documented that dopamine mimics IPC induced cardioprotection in normal rat hearts possibly through α_1_-adrenergic receptors [18]. Dopamine is also reported to affect the release of various mediators in pathological state like hyperlipidemia [19–22] which has been cited responsible for the blunted cardioprotective response of IPC [12, 13]. Therefore, the protection offered by dopamine in hyperlipidemic subjects can be ascertained (Fig. 1b). Therefore, the present investigation was proposed to examine the role of D2 signaling in cardioprotective effect of preconditioning in hyperlipidemic rat hearts.

## Methods

### Animals

Wistar albino rats of either sex weighing 160-180 g were obtained from the animal house, Shoolini University, Solan, HP, India (Reg. No. 1541/PO/a/11/CPCSEA) and maintained at 25 ± 2 °C temperature, 45 ± 5 % humidity, food and water *ad libitum*. The animals were allowed to acclimatize to the environment for 7 days prior to the experiment. The experiment was conducted in accordance with the guidelines of Committee for the purpose of control and supervision of experiments on animals (CPCSEA) and was approved by Institutional animal ethical committee (IAEC) vide ref. no. IAEC/SU-PHARM/12/016.

### Induction of hyperlipidemia (HL)

The animals were divided into 8 groups, each comprising eight animals (n = 8) except two groups which were fed on normal chow diet and comprising six animals in each group (n = 6). The animals were fed on high fat diet (HFD) for 4 weeks. The success rate of hyperlipidemia using HFD in Wistar rats is 80-90 %. High fat diet was prepared using following ingredients: Powdered Normal chow (365 g/Kg), Butter (310 g/Kg), Casein (250 g/Kg), Cholesterol (10 g/Kg), Vitamin and mineral mix (60 g/Kg), DL-Methionine (03 g/Kg), Yeast powder (01 g/Kg) and Sodium chloride (01 g/Kg) [23]

### Isolated heart preparation and perfusion protocol

After the induction of hyperlipidemia for 4 weeks, rats were administered with heparin (500 IU/Kg, *i.p.*) 20 min. prior to the experimentation. The animals were then sacrificed by cervical dislocation. Thorax was surgically opened; hearts were rapidly excised and placed immediately in cold perfusion buffer before being mounted on Langendorff’s apparatus. The isolated heart preparation on Langendorff’s apparatus was provided with retrograde perfusion of oxygenated (95 % O_2_ - 5 % CO_2_) normothermic (37 °C) Krebs-Henseleit (KH) buffer solution at a constant pressure of 100 cm H_2_O. The heart was then mounted over assembly within the time interval of 1 min after excision. The composition of KH buffer used was (g/L): 2.1 NaHCO_3_, 0.35 KCl, 0.28 NaCl, 1.28 MgSO_4_, 0.16 KH_2_PO_4_, 0.28 CaCl_2_ and 2.0 Glucose; (pH 7.4). The hearts were allowed to stabilize for 10 min before undergoing any treatment. The period of 30 min of ischemia followed by 120 min of reperfusion was used to induce ischemia-reperfusion injury.

### Experimental protocol

**Fig. 2:**
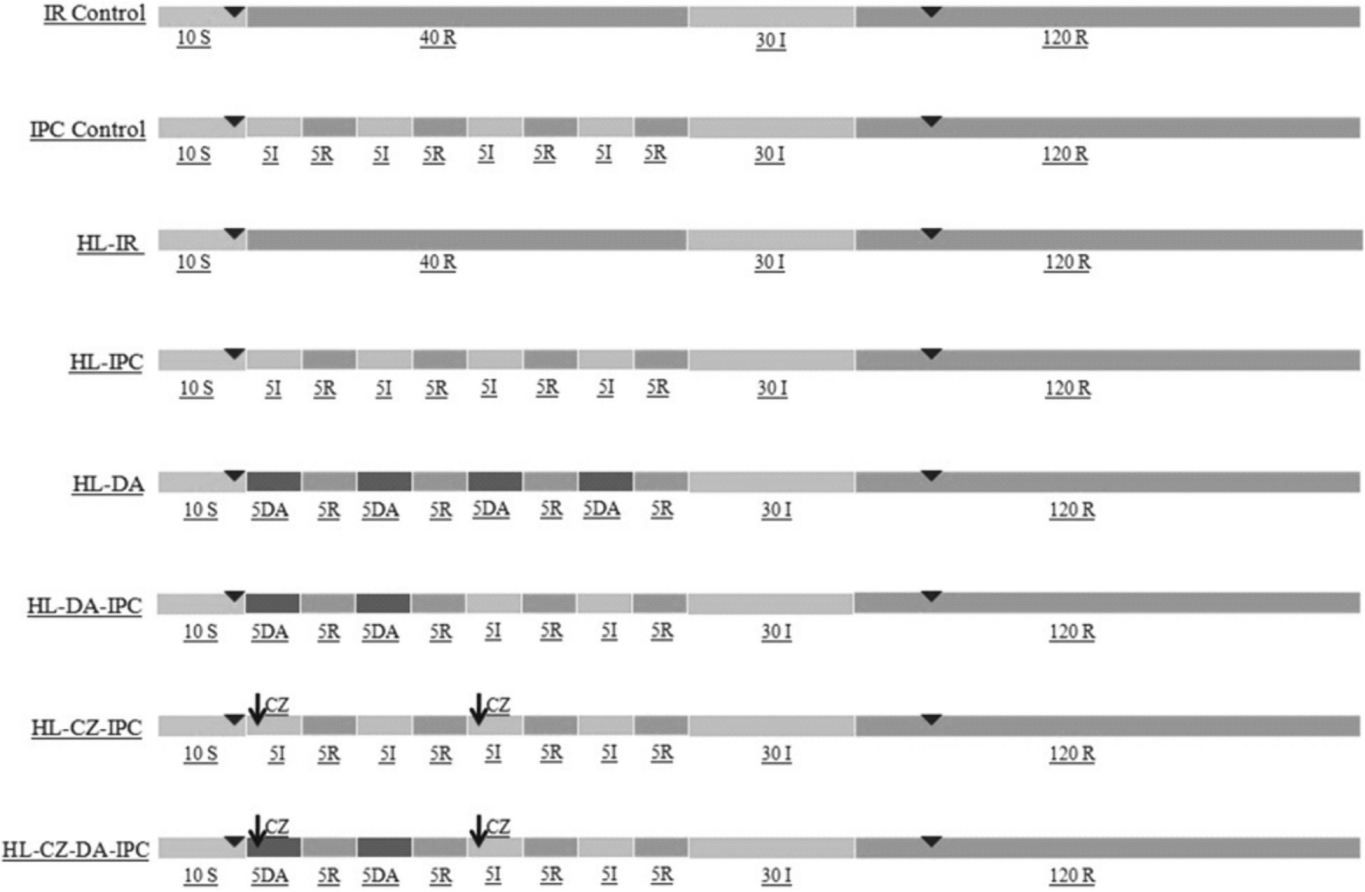
Experimental protocol: S- stabilization period, I- Ischemia, R- Reperfusion, DA- Dopamine, CZ- Clozapine, ▼- Perfusate collection

The animals were divided into 8 groups (*n* = 6) (Fig. 2). The first group (I/R control) was fed on normal chow diet and subjected to ischemia reperfusion injury. The second group (IPC control) was fed on normal chow diet and subjected to preconditioning episodes before ischemia reperfusion injury. The third group (HL-IR) was fed on HFD and subjected to ischemia reperfusion injury. The fourth group (HL-IPC) was fed on HFD and subjected to preconditioning episodes before ischemia reperfusion injury. The fifth group (HL-DA) was fed on HFD and subjected to dopamine preconditioning before ischemia reperfusion injury. The sixth group (HL-DA-IPC) was fed on HFD and subjected to both dopamine & ischemic preconditioning episodes before ischemia reperfusion injury. The seventh group (HL-CZ-IPC) was fed on HFD and subjected to clozapine treatment & preconditioning episodes before ischemia reperfusion injury. The eighth group (HL-CZ-DA-IPC) was fed on HFD and subjected to clozapine treatment, Dopamine & ischemic preconditioning episodes before the ischemia reperfusion injury.

### Pharmacological assessments

#### % change in body weight and serum lipid profile

After 4 weeks of HFD treatment, % change in body weight and level of serum lipid markers: total cholesterol, triglycerides, LDL, HDL and VLDL were estimated as an index of hyperlipidemia using biochemical kits from Transasia Bio-medical Ltd., Baddi, India.

#### Measurement of coronary LDH and CKMB

Coronary effluent was collected after the stabilization period, and during first 5 min of period of reperfusion and the level of lactate dehydrogenase (LDH) and creatine kinase MB (CKMB) were estimated using biochemical kits from Transasia Bio-medical Ltd., Baddi, India.

#### Hemodynamic parameters

Coronary flow Rate (CFR) was assessed as a parameter for cardiac function by collecting the coronary effluent during the stabilization period and at the end of 120 min of reperfusion. It was expressed in mL/min.

#### Infarct size measurement

After the experiment, the hearts were removed and frozen to facilitate slicing into 2 mm thick transverse sections across the long axis. The slices were incubated in 1 % triphenyl tetrazolium chloride (TTC) in phosphate buffer (pH 7.4) for 30 min at 37 °C. The slices were then immersed in 10 % formalin solution to enhance the contrast between unstained and stained cardiac tissue. The stained slices were observed for cardiac injury. The tissue stained brick red was considered viable and pale coloured tissue was considered as damaged. The slices were photographed and infarcted area was calculated by planimetry using ‘ImageJ’ (version 1.47) software from the National Institute of Health (NIH), USA. Infarct size was expressed as a percentage of left ventricular volume.

#### Statistical analysis

Results were expressed as mean ± SD analysed by Student t-test (for body weight and lipid profile), One-way analysis of variance (ANOVA) (for infarct area) and Two way analysis of variance (ANOVA) (for LDH, CKMB and CFR) followed by Bonferroni’s multiple comparison test as *post hoc* analysis to determine statistical difference between groups using ‘GraphPad Prism’ (version-6.02) software. *P* value < 0.05 was considered to be statistically significant.

## Results

### Effect of high fat diet treatment on body weight

**Fig. 3:**
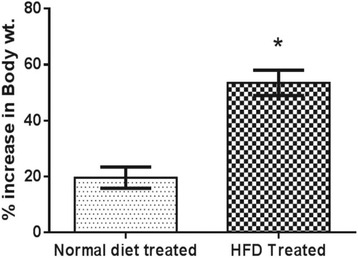
Effect of high fat diet treatment on Body weight; (*n* = 12 for normal diet and *n* = 36 for HFD) Results: Mean ± SD; analyzed by Student t-test; *p < 0.05 vs. Normal diet treated

Treatment with high fat diet for 4 weeks produced significant elevation (*p* < 0.05) in % increase in body weight in HFD treated animals (53.52 ± 4.54) as compared to animals treated with normal chow diet (19.63 ± 3.78) (Fig. 3).

### Effect of high fat diet treatment on Lipid profile

#### Total cholesterol and Triglycerides

**Fig. 4:**
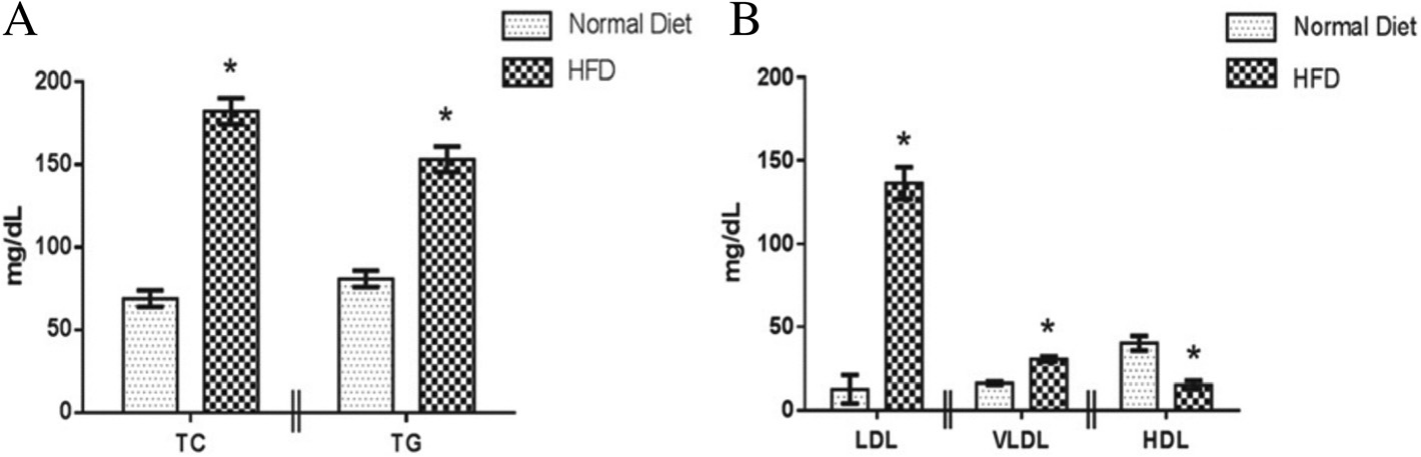
**(a)** Effect of high fat diet treatment on Total cholesterol and Triglycerides; (*n* = 12 for normal diet and *n* = 36 for HFD) **(b)** Effect of high fat diet treatment on LDL, VLDL and HDL; (*n* = 12 for normal diet and *n* = 36 for HFD); Results: Mean ± SD; analyzed by Student t-test; *p < 0.05 vs. Normal diet treated

Treatment with high fat diet produced significant increase (p < 0.05) in the levels of total cholesterol and triglycerides in the HFD treated animals (182.39 ± 7.9 and 153.16 ± 7.7 respectively) as compared to animals treated with normal chow diet (68.91 ± 5.05 and 80.96 ± 4.96 respectively) (Fig. 4a).

#### LDL, VLDL and HDL

Treatment with high fat diet produced a significant increase (*p* < 0.05) in the serum levels of LDL and VLDL whereas a significant decrease (*p* < 0.05) in the levels of HDL was observed in the HFD treated animals (136.46 ± 9.61, 30.63 ± 1.54 and 15.29 ± 2.52 respectively) as compared to normal diet treated animals (12.54 ± 8.46, 16.19 ± 0.99 and 40.17 ± 4.41 respectively) (Fig. 4b).

### Effect of preconditioning on cardiac enzymes

#### Lactate Dehydrogenase (LDH)

**Fig. 5:**
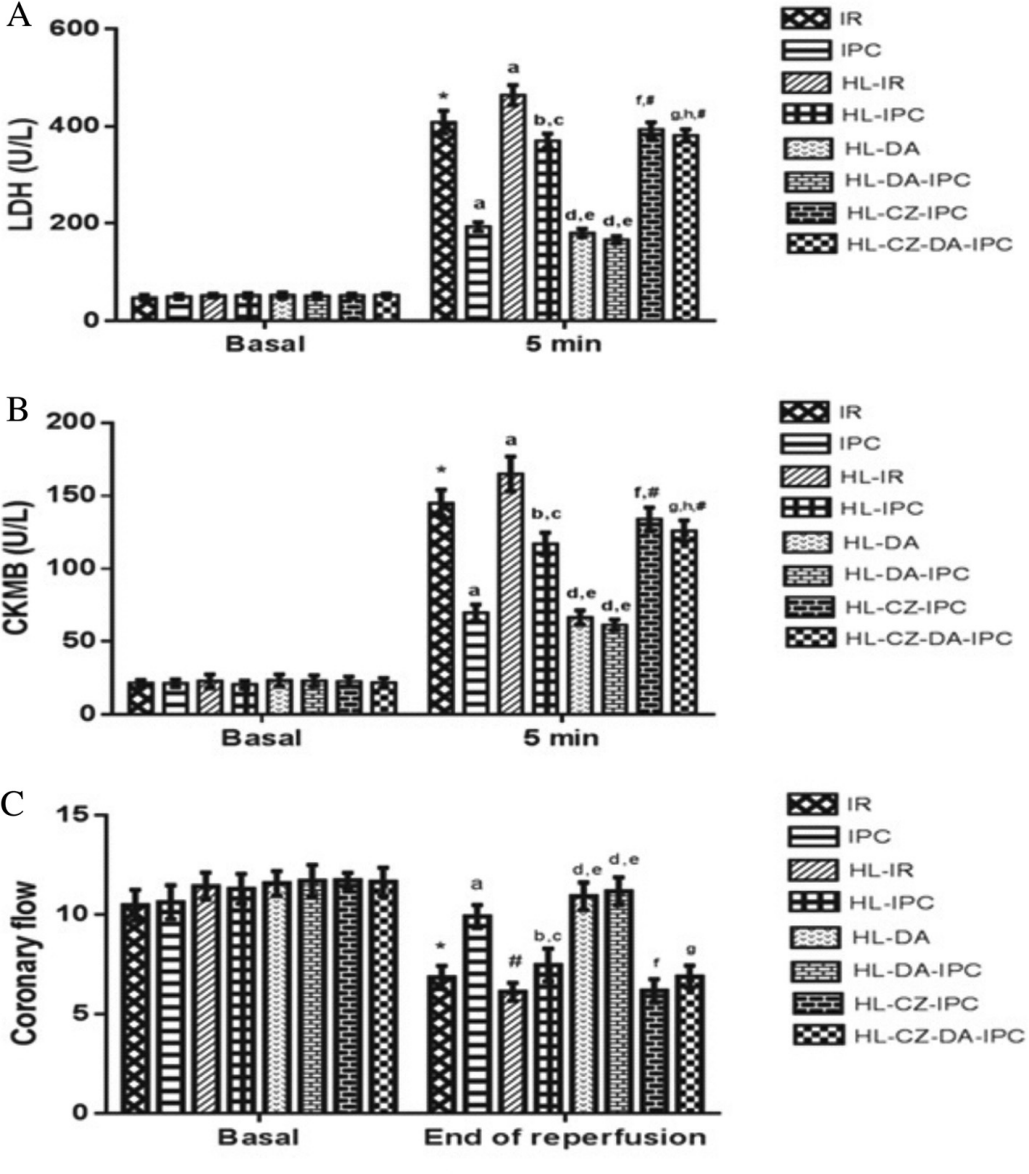
Effect of preconditioning on **(a)** Lactate Dehydrogenase (LDH), **(b)** Creatine Kinase (CK-MB) and **(c)** Coronary flow rate (CFR); (*n* = 6) Results: Mean ± SD; analyzed by Two-way ANOVA followed by Bonferroni’s multiple comparison test as *post hoc* analysis; ^*^ *p* < 0.05 vs. Basal, ^a^ *p* < 0.05 vs. IR control, ^b^ *p* < 0.05 vs. HL-IR, ^c^ *p* < 0.05 vs. IPC control, ^d^ *p* < 0.05 vs. HL-IR, ^e^ *p* < 0.05 vs. HL-IPC, ^f^ *p* < 0.05 vs. HL-IPC, ^g^ *p* < 0.05 vs. HL-DA-IPC and ^#^ *p* < 0.05 vs. HL-IR were significant and ^h^ *p* > 0.05 vs. HL-CZ-IPC was insignificant

Induction with ischemia-reperfusion produced significant increase (*p* < 0.05) in LDH level (408.69 ± 23.66), as compared to its basal value (47.50 ± 5.27). IPC group showed significant decrease (*p* < 0.05), whereas HL-IR group showed significant increase (*p* < 0.05) in LDH levels (193.32 ± 9.03 and 463.96 ± 20.18 respectively), as compared to IR treated group (408.69 ± 23.66). HL-IPC group showed significant decrease in LDH level (370.30 ± 14.70), as compared to HL-IR group (463.96 ± 20.18) and a significant increase, as compared to IPC (193.32 ± 9.03)(*p* < 0.05). However, HL-DA and HL-DA-IPC groups showed significant decrease (*p* < 0.05) in LDH levels (180.30 ± 8.47 and 166.05 ± 7.81 respectively), as compared to both HL-IR (463.96 ± 20.18) and HL-IPC groups (370.30 ± 14.70). A significant elevation in LDH level was also observed in HL-CZ-IPC (393.85 ± 14.46), as compared to HL-IPC group (370.30 ± 14.70) (*p* < 0.05). HL-CZ-DA-IPC group also produced a significant increase (*p* < 0.05) in LDH level (381.43 ± 12.47), as compared to HL-DA-IPC group (166.05 ± 7.81), however the reduction in LDH levels in HL-CZ-DA-IPC group (381.43 ± 12.47), as compared to HL-CZ-IPC (393.85 ± 14.46) was insignificant (*p* > 0.05). The levels of LDH in HL-CZ-IPC and HL-CZ-DA-IPC groups were also found to be significantly lesser (*p* < 0.05), as compared to HL-IR (Fig. 5a).

#### Creatine Kinase (CK-MB)

Induction with ischemia-reperfusion produced significant increase (*p* < 0.05) in CKMB level (145.12 ± 8.89) as compared to its basal value (21.57 ± 1.95). IPC group showed significant decrease (*p* < 0.05), whereas HL-IR group showed significant increase (*p* < 0.05) in CKMB level (69.74 ± 5.66 and 164.92 ± 11.82 respectively), as compared to IR treated group (145.12 ± 8.89). HL-IPC group showed significant decrease in CKMB levels (117.12 ± 7.64), as compared to HL-IR group (164.92 ± 11.82) and a significant increase, as compared to IPC (69.74 ± 5.66)(*p* < 0.05). However, HL-DA and HL-DA-IPC groups showed significant decrease (*p* < 0.05) in the levels of CKMB (66.51 ± 4.89 and 61.48 ± 3.64 respectively), as compared to both HL-IR (164.92 ± 11.82) and HL-IPC groups (117.12 ± 7.64). A significant elevation in CKMB levels was also observed in HL-CZ-IPC group (134.01 ± 8.05), as compared to HL-IPC (117.12 ± 7.64) (*p* < 0.05). HL-CZ-DA-IPC group also showed a significant increase (*p* < 0.05) in CKMB level (126.13 ± 7.12), as compared to HL-DA-IPC (61.48 ± 3.64), however the reduction in CKMB levels in HL-CZ-DA-IPC group (126.13 ± 7.12), as compared to HL-CZ-IPC (134.01 ± 8.05) was insignificant (*p* > 0.05). The levels of CKMB in HL-CZ-IPC and HL-CZ-DA-IPC groups were also found to be significantly lesser (*p* < 0.05), as compared to HL-IR (Fig. 5b).

#### Coronary flow rate (CFR)

Induction with ischemia-reperfusion in normal (IR) and hyperlipidemic hearts (HL-IR) showed significant decrease (*p* < 0.05) in coronary flow rate levels (6.85 ± 0.59 and 6.11 ± 0.45 respectively), as compared to their basal values (10.48 ± 0.78 and 11.43 ± 0.67 respectively). However, a significant increase in CFR of IPC (9.91 ± 0.57), as compared to IR (6.85 ± 0.59) and in CFR of HL-IPC (7.46 ± 0.83), as compared to HL-IR (6.11 ± 0.45) were observed (*p* < 0.05). HL-DA and HL-DA-IPC groups also showed significant increase in CFR levels (10.93 ± 0.69 and 11.18 ± 0.69 respectively), as compared to HL-IR (6.11 ± 0.45) and HL-IPC groups (7.46 ± 0.83) (*p* < 0.05). A significant decrease (*p* < 0.05) in coronary flow rate was observed in HL-CZ-IPC group (6.16 ± 0.59), as compared to HL-IPC (7.46 ± 0.83) and in HL-CZ-DA-IPC group (6.88 ± 0.56), as compared to HL-DA-IPC (11.18 ± 0.69) (Fig. 5c).

### Effect of preconditioning on infarct size

**Fig. 6:**
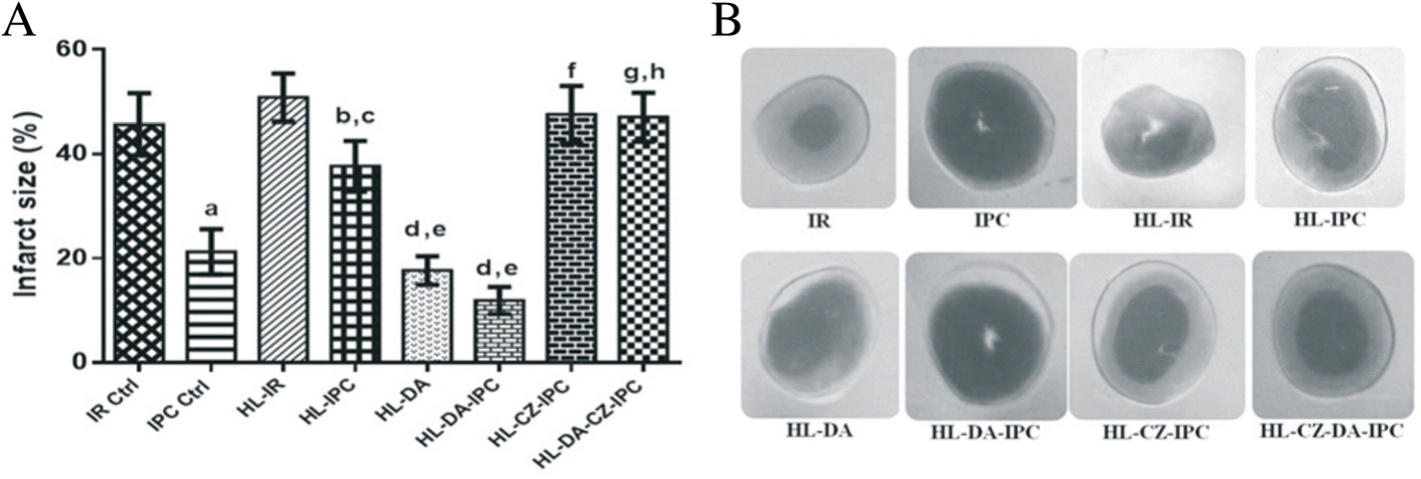
**(a)** Effect of preconditioning on Infarct Size; (*n* = 6) Results: Mean ± SD; analyzed by One-way ANOVA followed by Bonferroni’s multiple comparison test as *post hoc* analysis; ^a^ *p* < 0.05 vs. IR control, ^b^ *p* < 0.05 vs. HL-IR, ^c^ *p* < 0.05 vs. IPC control, ^d^ *p* < 0.05 vs. HL-IR, ^e^ *p* < 0.05 vs. HL-IPC, ^f^ *p* < 0.05 vs. HL-IPC, ^g^ *p* < 0.05 vs. HL-DA-IPC were significant and ^h^ *p* > 0.05 vs. HL-CZ-IPC was insignificant. **(b)** Effect of Preconditioning on Infarct Size

Treatment with IPC produced a significant decrease (*p* < 0.05) in infarct size (21.22 ± 4.36) as compared to IR treated group (45.64 ± 5.93). HL-IPC group showed significant decrease in infarct size (37.65 ± 4.88) as compared to HL-IR group (50.78 ± 4.62) and a significant increase as compared to IPC (21.22 ± 4.36) (*p* < 0.05). However, HL-DA and HL-DA-IPC groups showed significant fall (*p* < 0.05) in the infarct size (17.66 ± 2.73 and 11.87 ± 2.61 respectively) as compared to both HL-IR (50.78 ± 4.62) and HL-IPC groups (37.65 ± 4.88). A significant rise in infarct size was also produced in HL-CZ-IPC group (47.54 ± 5.45) as compared to HL-IPC (37.65 ± 4.88) (*p* < 0.05). HL-CZ-DA-IPC group also produced a significant increase (*p* < 0.05) in infarct size (47.04 ± 4.66) as compared to HL-DA-IPC (11.87 ± 2.61), however the fall in infarct size in HL-CZ-DA-IPC group (47.04 ± 4.66) as compared to HL-CZ-IPC (47.54 ± 5.45) was insignificant (*p* > 0.05) (Fig. 6a & b).

## Discussion

Ischemic preconditioning (IPC) is a well known phenomenon which limits the infarct size and offers cardioprotection to myocardium [24]. Primarily, it was observed that four cycles of 5 min ischemia with intermittent reperfusion was able to limit infarct size significantly by 75 % on subjection to subsequent prolonged ischemic insult [1]. With the advent of time, various attempts have been made to identify underlying endogenous mechanisms of preconditioning and also to mimic its cardioprotective effects by some pharmacological agents, clinically referred as pharmacological preconditioning [25].

Hyperlipidemia subsequently results in atherosclerosis, coronary heart disease, hypertension, myocardial infarction and stroke. The elevated levels of serum lipids, TGs, clogged arteries and release of endogenous cell mediators in hyperlipidemic state exaggerate the myocardial ischemic injury [12]. The cardioprotective effect of preconditioning is blunted in hyperlipidemic condition and is one of the major limiting factors for the efficacy of IPC clinically [11].

Dopamine is reported to offer cardioprotection by pharmacological preconditioning in normal rat hearts via α_1_ receptors [18]. Interestingly, activation of dopamine D2 receptors *in-vitro* has been found to inhibit apoptosis of cardiomyocytes encountered during ischemia/reperfusion through various pathways and also the expression of D2 receptor was reported to increase during ischemia/ reperfusion [17]. Dopamine is also reported to regulate release of various mediators in hyperlipidemic condition [19–22] that are responsible for the blunted cardioprotective effect of preconditioning [12, 13]. Therefore, it becomes important to explore whether D2 receptors have a role in ischemic preconditioning and also that whether the blunted cardioprotective effect can be restored in hyperlipidemic rat hearts by dopamine induced pharmacological preconditioning.

In the present study, treatment with high fat diet induced hyperlipidemia in the animals, characterized by significantly elevated LDL, VLDL, TC and TG with reduced HDL levels and also increased body weights after 4 weeks which conforms to previously reported studies [23]. Ischemic preconditioning provided cardioprotection to normal rat hearts against ischemia-reperfusion, as evidenced by significant decrease in infarct size, LDH and CKMB, in comparison to I/R control [26].

The attenuated cardioprotective effect of preconditioning in hyperlipidemic condition was also reproduced against I/R injury in the present study as evidenced by increased level of LDH, CKMB and infarct size in hyperlipidemic preconditioned rat hearts, as compared to IPC treated normal rat hearts [26]. This effect was possibly due to the release of various mediators (eNOS, nucleotidase, peroxynitrite, Tetrahydrobiopterin, superoxide anion etc.) in hyperlipidemic state which affect the preconditioning cascade as suggested in previous studies [13]. Pharmacological preconditioning using dopamine was found to reduce the infarct size, LDH and CKMB levels in hyperlipidemic rat hearts, as compared to hyperlipidemic and preconditioned hyperlipidemic rat hearts. Clozapine, a D2 receptor antagonist was found to abort cardioprotection as the levels of infarct size, LDH and CKMB were significantly increased in clozapine treated preconditioned hyperlipidemic hearts, as compared to ischemic preconditioning treated hyperlipidemic rat hearts.

A combined cardioprotective effect of ischemic preconditioning and dopamine preconditioning was also explored in the hyperlipidemic hearts and the levels of LDH, CKMB and infarct size were found to be significantly decreased as compared to non-preconditioned and preconditioned hyperlipidemic hearts. This effect was further abolished with the use of clozapine administration as the infarct size, LDH and CKMB were significantly elevated. This confirms the availability of dopamin and role of D2 signaling in both ischemic preconditioning and dopamine induced pharmacological preconditioning in the pathological state of hyperlipidemia.

The coronary flow rate (CFR) was found to be decreased against I/R in both normal and hyperlipidemic rat hearts, as compared to basal values. CFR also decreased in preconditioned hyperlipidemic hearts and clozapine treated preconditioned hearts. The levels of CFR were restored in groups treated with ischemic preconditioning alone and dopamine preconditioned groups with or without ischemic preconditioning, as compared to normal rat hearts and ischemic preconditioned hyperlipidemic hearts respectively.

Therefore, the findings may conclude for the involvement of D2 signalling in ischemic preconditioning and dopamine induced pharmacological preconditioning in hyperlipidemic rat hearts. Further, research can be targeted on the role of pharmacological preconditioning in other pathological states like Diabetes or in aged subjects for it to be rendered clinically applicable.
